# Risk factors of recurrent thyroid nodules after radiofrequency ablation

**DOI:** 10.4314/ahs.v23i3.68

**Published:** 2023-09

**Authors:** Yuke Xia, Yuehe Fu, Mengjia Qian, Yiyao Cui

**Affiliations:** Department of Thyroid and Breast Surgery, The Affiliated Jiangning Hospital of Nanjing Medical University, 211100, Nanjing, China

**Keywords:** Thyroid nodules, radiofrequency ablation, recurrence, risk factors

## Abstract

**Objective:**

To investigate the risk factors of thyroid nodule recurrence after radiofrequency ablation (RFA). METHODS The medical record information of 120 patients with thyroid nodules admitted to our hospital from June 2019 to April 2022 was retrospectively analysed. All participants received RFA treatment. According to the results of the postoperative thyroid ultrasound examination (USG), the patients were divided into the recurrence group (R, N=16) and the non-recurrence group (NR, N=104). Binary logistic regression analysis was performed to identify the independent risk factors of thyroid nodule recurrence after RFA. The receiver operating characteristic (ROC) curve was used to analyse the value of the forecast of each independent factor and combined model for thyroid nodule recurrence after RFA.

**Results:**

During the follow-up period, 16 patients recurred, and the recurrence rate was 13.33%. Univariate regression analysis showed that whether the nodules are solitary (WNS), nodule diameter (ND), the degree of risk of nodular location (DRN), recurrent laryngeal nerve (RLN) injury were associated with thyroid nodule recurrence after RFA (P<0.05). Binary logistic regression analysis showed that WNS, ND, DRN and RLN injury were independent risk factors for the recurrence of thyroid nodules after RFA (P<0.05). ROC analysis of independent factors and combined model showed that solitary nodules, nodule diameter and nodule location risk degree had diagnostic value, while RLN injury had no predictive value. The combined model is more predictive than the independent factors. Conclusions: The risk factors of recurrent thyroid nodules after radiofrequency ablation are related to WNS, ND, DRN and so on, which should be paid attention to and preventive measures should be taken.

## Introduction

A thyroid nodule is a mass inside the thyroid gland that moves up and down with the thyroid gland during swallowing. The disease is very common in clinical treatment, and the incidence of women is significantly higher than that of men. It is shown that 90% of the detected nodules are benign nodules 1, 2. Most benign nodules can be followed up without treatment. When the symptoms of neck compression, combined with hyperthyroidism and medical treatment ineffective or malignant tendency, surgical resection is the traditional treatment 3. However, the disadvantages of total thyroidectomy are very obvious. This type of surgery can cause great trauma to the patient's body in the process of treatment. The postoperative scar is easy to leave, not only slowing down the postoperative recovery of patients but also affecting the appearance. Normal thyroid glands may be damaged during the operation, resulting in adverse effects of treatment 4. In addition, the residual gland recurrence rate is still high after total or partial thyroidectomy or nodule excision. And it is prone to different degrees of hypothyroidism complications 5.

Ultrasound-guided RFA is gradually applied to patients with recurrent thyroid nodules and thyroid cancer after surgery. Under the guidance of ultrasound, high-frequency alternating current is generated through radiofrequency electrodes, which cause polar molecules and electrons in the tissue to oscillate at high speed, thereby generating heat 6. When the temperature approaches 50 °C, the protein solidifies, leading to necrosis of the tumor cells and thus elimination of the tumor 7. This surgical method has the advantages of small trauma, quick recovery, high safety and good efficacy, so it is often used in the clinical treatment of tumors and nodules 6, 8.

The recurrence rate of thyroid nodules after surgery is high. Studies in China reported that the recurrence rate was 18-30% 9, while other countries reported that the recurrence rate was 10-45% 10, and most of them recurred within 5 years after surgery. The high recurrence rate harms the prognosis of patients. To further understand the risk factors affecting their recurrence, we analysed 120 patients with thyroid nodules who underwent radiofrequency ablation treatment at our hospital between June 2019 and April 2022. They were followed up after RFA treatment and compared the clinical data of patients with and without recurrence after surgery. To provide a clinical reference for ultrasound-guided RFA in the treatment of thyroid nodules.

## Methods

### Patients

A total of 120 patients with thyroid nodules admitted to our hospital from June 2019 to April 2022 were included in this study. All participants were treated with RFA after admission.

**Inclusion criteria:** (1) All patients showed obvious clinical symptoms and thyroid nodules were found during physical examination or palpation; (2) Preoperative histological biopsy showed benign nodules; (3) Preoperative thyroid function was normal; (4) The coagulation function was normal and the liver and kidney functions were normal. (5) The treatment cooperation of the patients was good, and the patients and their relatives were aware and agree and signed informed consent.

**Exclusion criteria:** (1) Patients with vital organ dysfunction, including cardiopulmonary function, liver and kidney function impairment; (2) Previous history of neck surgery or radiotherapy; (3) Patients with poor compliance without regular follow-up in our hospital.

### Follow-up

The thyroid function and ultrasound examination (USG) were reviewed every 3 months after the operation. If the condition is relatively stable for more than 1 year, it can be reviewed every 6 months. The starting point of follow-up was the operation time of the patient, and the end point of follow-up was the recurrence of the patient or the last follow-up time (July 2022). Recurrence was defined as a USG-suggestive nodule more than 3 months after initial treatment. Data were collected including gender, age, and grouping according to recurrence.

### Statistical analysis

Statistical analysis was conducted by SPSS version 23 software. The continuous variables were displayed as mean ± standard deviation. Count variables were represented by n (%). A Student's t-test and χ^2^ test were performed to evaluate the correlation between demographic and preoperative and postoperative characteristics. Binary logistic regression was used to analyse the risk factors of recurrent thyroid nodules after RFA treatment. Univariate analysis was used to obtain meaningful variables, and then the meaningful variables were included in binary regression model analysis to screen out the independent risk factors of recurrent thyroid nodules after RFA. The ability of each independent risk factor and combined model to predict thyroid recurrence was analysed by receiver Operating characteristic curve (ROC) analysis tool of SPSS version 23 software. The ROC curve is a curve reflecting the relationship between sensitivity and specificity. Area Under Curve (AUC), which is used to represent the prediction accuracy. The AUC value is proportional to the accuracy. It is generally believed that 0.5 <AUC≤0.7 suggests poor prediction ability, 0.7 <AUC≤0.9 implies better predictive ability, and AUC> 0.9 indicates high predictive value. P <0.05 was considered a statistically significant difference. /span>

## Results

The male (n=31, 25.83%) to female (n=89, 74.17%) ratio is about 1:3, with an average age of 52.55±7.03 years, ranging from 35 to 72 years. There were 48 patients with multiple nodules and 72 patients with solitary nodules, with a total of 148 nodules. There were 95 solid nodules and 53 cystic nodules, with an average diameter of 1.94±0.43cm (1.06-2.99 cm). The number of cystic and solid nodules was 39 and 81, respectively. Up to the last follow-up in July 2022, the follow-up time was 3-35 months. During the follow-up period, 16 cases (13.33%) had a recurrence. The demographic characteristics of the recurrence group (R) and the non-recurrence group (NR) were compared. As shown in Table 1, statistical differences were found in the proportion of whether the nodules are solitary (WNS), nodule diameter (ND), nodule components, the degree of risk of nodular location (DRN) and whether recurrent laryngeal nerve (RLN) injury occurred in the two groups (P<0.05). No significant differences were found in gender, age and aspects between the two groups (P>0.05). Table 2 shows the preoperative thyroid function test showed that the patients were normal, and the FT3, FT4 increased slightly at 6 months after the operation, but there was no statistical significance between R and NR (P>0.05).

**Table 1:** Demographic characteristics

Variable	Total (n=120)	NR(n=104)	R(n=16)	*χ^2^*	P
Gender					
Male	31(25.83%)	29(27.88%)	2(12.50%)	1.004	0.316
Female	89(74.17%)	75(72.12%)	14(87.50%)		
Age(year)					
<60	103(85.83%)	90(86.54%)	13(81.25%)	0.032	0.857
≥60	17(14.17%)	14(13.46%)	3(18.75%)		
Solitary nodules					
No	32(26.67%)	22(21.15%)	10(62.50%)	3.894	0.048
Yes	88(73.33%)	82(78.85%)	6(37.50%)		
Average diameter of nodules (CM)					
<2	74(61.67%)	69(66.35%)	5(31.25%)	7.225	0.007
≥2	46(38.33%)	35(33.65%)	11(68.75%)		
Nodule component					
solid component <20%	41(34.17%)	32(30.77%)	9(56.25%)	4.003	0.045
solid component ≥20%	79(65.83)	72(69.23%)	7(43.75%)		
Risk of nodular location					
Low risk	96(80.00%)	90(86.54%)	6(37.50%)	20.841	<0.001
High risk	24(20.00%)	14(13.46%)	10(62.50%)		
Recurrent laryngeal nerve injury					
No	106(88.33%)	96(92.31%)	10(62.50%)	11.955	0.001
Yes	14(11.67%)	8(7.69%)	6(37.50%)		

**Table 2:** Preoperative and postoperative thyroid function

Measurements		Total (n=120)	NR(n=104)	R(n=16)	t	P
Preoperative thyroid function	FT3 (pmol/L)	4.87±0.52	4.86±0.54	4.93±0.41	-0.512	0.610
	FT4 (pmol/L)	16.51±1.82	16.17±1.22	15.55±1.39	1.843	0.068
	TSH (mU/L)	1.78±0.57	2.24±0.63	1.96±0.76	1.585	0.116
Thyroid function at 6 months after surgery	FT3 (pmol/L)	5.11±0.71	5.07±0.7	5.33±0.78	0.176	0.176
	FT4 (pmol/L)	16.82±2.21	16.88±2.19	16.36±2.4	0.875	0.383
	TSH (mU/L)	1.85±0.65	1.85±0.67	1.82±0.48	0.166	0.868

Of the 16 recurrent patients, 10 (62.5%) had nodules in high-risk locations, compared with 14 (13.46%) in the NR group. The incidence of RLN injury in the NR group was 8.51%, and all 6 cases were unilateral RLN injury. Among them, 4 cases presented with choking in drinking water without treatment, which improved spontaneously one week after operation, and the other 4 cases presented with hoarseness, which recovered to normal 5 days after nutritional nerve treatment. The incidence of postoperative RLN injury in group R was 37.5%, and all 6 cases were unilateral RLN injuries, manifested as postoperative hoarseness, which was treated with nutritional nerve therapy and returned to normal on the fifth day after operation.

A binary logistic regression model was established, with all patients as samples and recurrence as the dependent variable, with 1= recurrence and 0= no recurrence. The independent variables were gender, age, WNS, ND≥2cm, nodule composition, DRN, and RLN injury, and the assignment table was shown in Table 3. The input method was used for univariate regression analysis, and the results are shown in Table 4. WNS, ND, DRN and RLN injury were risk factors for thyroid nodule recurrence (P<0.05). There is no statistical difference between thyroid nodule reoccurrence and gender, age ≥60 years, and nodule composition (P>0.05).

**Table 3:** The assignment of variables

Variable	Assignment
Whether recurrence	No=0, Yes=1
Gender	Male=0, Female=1
Age(year)	<60 =0, ≥60 =1
Solitary nodules	No=0, Yes=1
Average diameter of nodules (CM)	<2=0, ≥2=1
Nodule component	solid component <20%=0, solid component ≥20%=1
Risk of nodular location	Low risk=0, High risk=1
Recurrent laryngeal nerve injury	No=0, Yes=1

**Table 4:** Univariate logistic regression analysis

Variable	β	Standard error	Wald*χ^2^*	P	95%CI
Gender	0.996	0.787	1.601	0.206	2.707(0.579,12.655)
Age	0.394	0.702	0.316	0.574	1.484(0.375,5.873)
Solitary nodules	-1.827	0.569	10.287	0.001	0.161(0.053,0.491)
Average diameter of nodules (CM)	1.467	0.578	6.446	0.011	4.337(1.397,13.462)
Nodule component	-1.062	0.547	3.772	0.052	0.346(0.118,1.01)
Risk of nodular location	2.372	0.591	16.106	<0.001	10.714(3.365,34.117)
Recurrent laryngeal nerve injury	1.974	0.634	9.692	0.002	7.200(2.078,24.951)

According to the results of univariate regression analysis, WNS, ND, DRN and RLN injuries were included in the binary regression model. The analysis results were displayed in Table 5. WNS, ND, DRN and RLN injuries were independent risk factors for thyroid nodule recurrence (P<0.05). Hosmer-Lemeshow goodness of fit test showed that X^2^ =5.575, P=0.233, indicating good fit and high reliability of the model.

**Table 5:** Binary logistic regression analysis

Variable	β	Standard error	Wald*χ^2^*	P	95%CI
Solitary nodules	-2.757	0.935	8.689	0.003	0.063(0.010,0.397)
Average diameter of nodules	1.746	0.821	4.525	0.033	5.732(1.147,28.641)
Risk of nodular location	3.793	1.051	13.022	<0.001	44.391(5.657,348.337)
Recurrent laryngeal nerve injury	3.928	1.224	10.303	0.001	50.806(4.616,559.172)

**Figure 1:**
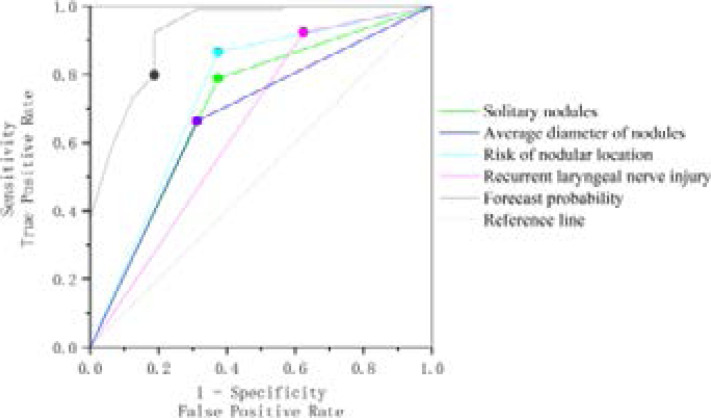
Receiver operating curves of independent factors and combined model

ROC curve analysis of the above independent risk factors and their combined model showed that the AUC of each factor was 0.707 for WNS, 0.675 for ND, 0.745 for DRN, and 0.924 for combined model (95%CI: 0.847-1.000), with statistical significance (P<0.05). The AUC of RLN injury was 0.649, and there was no statistical significance (P>0.05). ND was greater than 0.5, but less than 0.7 (P=0.024), indicating that ND has a significant diagnostic value for thyroid nodule recurrence after RFA, but its diagnostic value is still low. The combined value of independent risk factors was higher than that of single risk factors. It indicates that the model has good discrimination and high diagnostic value, as shown in Figure 1 and Table 6.

**Table 6:** Receiver operating curve and area under curve (AUC)

Test result variable	AUC	P	Sensitivity	Specificity	95%CI
Solitary nodules	0.707	0.008	0.625	0.788	(0.560,0.854)
Average diameter of nodules	0.675	0.024	0.688	0.663	(0.534,0.817)
Risk of nodular location	0.745	0.002	0.625	0.865	(0.598,0.892)
Recurrent laryngeal nerve injury	0.649	0.056	0.375	0.923	(0.486,0.813)
Forecast probability	0.924	<0.001	0.813	0.923	(0.847,1.000)

## Discussion

Although thyroid nodules are mostly benign lesions, nodules will affect the aesthetics of patients, and the large volume of nodules will cause local compression. Therefore, patients with thyroid nodules need to be treated effectively as early as possible to improve their quality of life. At present, RFA therapy is mainly used in the clinical treatment of tumor lesions. It has been reported that the 5-year recurrence rate of thyroid RFA is 20%-23%^11^,^12^. Its pathogenesis is diffuse thyroid hyperplasia and enlargement caused by long-term iodine deficiency, and long-term proliferative and degenerative lesions alternate repeatedly, leading to nodules. However, in a small number of cases, nodule regrowth is caused by an underlying malignant tumor 13. Li et al. 14 reported that RFA had a low effective rate in the treatment in children, with a volume reduction rate (VPR) of 77.5% and a nodule regeneration rate of 22.9% at 12 months after operation. Du et al. 15 applied microwave ablation in patients with thyroid nodules and found that nodules gradually decreased with time through regular follow-up, and the VPR of nodules was 96.9% at 48 months after operation. Although the efficacy of RFA is good, postoperative disease recurrence can affect the efficacy of treatment 6.

Due to the relatively complex relationship between the tissues around the thyroid gland, there are many blood vessels and nerves distributed, which increases the risk of surgery to a certain extent. The use of expanded ablation treatment is prone to cause thermal damage to the blood vessels and nerves of patients, resulting in articulation disorders, nodule rupture and other complications 16,17. The incidence of major complications in the application of RFA in the thyroid nodule has been reported to be as high as 3.8% 18, and another study of RFA applied to the thyroid nodules in children reported complication rates as high as 4.8% and found higher VPR in nodules with a predominantly cystic component 14. Therefore, it makes sense explore the risk factors of thyroid nodule recurrence after RFA and take corresponding intervention measures to improve the prognosis of the disease.

Our analysis showed that WNS, ND, DRN and RLN injury were all risk factors for the recurrence of thyroid nodules after RFA. Although there is no correlation between solitary or multiple nodules and whether they are benign or malignant, solitary nodules are an important prognostic factor. One study found a high recurrence rate after lobectomy for solitary nodules 19. However, the internal mechanism of solitary nodules and their long-term prognosis has not been reported. We speculate that it may be related to the pathogenesis of nodules. In addition, the recurrence of nodules was associated with the type of surgery and the duration of follow-up 20. Cesareo et al. 21 reported that nodule volume may be an important predictor of the efficacy of RFA in the clinical application of thyroid nodules. VPR of moderate nodules (>12ml) was significantly lower than that of the small nodule (>12ml), and the former had a higher risk of recurrence. Thyroid nodules usually show slow progression and slow growth rate, but the risk of postoperative recurrence is high, the difficulty of the second operation is increased, and the complications are also increased 22. The larger the nodule diameter, the more complex the relationship between the nodule and the surrounding organs and blood vessels, which undoubtedly increases the risk of postoperative complications 23. The adjacency of thyroid gland is complicated, and the distribution of blood vessels and nerves should be fully considered during the ablation. Therefore, for multiple nodules with large diameters, incomplete elimination may exist, which is one of the important reasons for the recurrence of thyroid nodules 24. In addition, nodular vasculature is significantly associated with thyroid nodule recurrence, and when nodules are visible or develop into vasculature, they have considerable regenerative potential 25, 26.

Zhao et al. 27 reported that RLN injury were associated with nodule diameter and the distance from nodule to thyroid sac. Poor recognition of recurrent laryngeal nerve and involuntary nerve excision during operation are the main causes of RLN injury 28. Of course, these factors are related to nodule diameter, location and other factors. The occurrence of complications is related to the clinician's knowledge and manipulation, and the patient's condition. Whether the clinician is familiar with neck anatomy, ultrasound image interpretation, ablation power adjustment and ablation time control will affect the incidence of complications 29.

Whether a thyroid nodule needs surgical resection should be decided according to the size, nature and other factors of the nodule. RFA is suitable for the nodules to be benign and small, otherwise surgical resection may be required. The former can eliminate thyroid nodules under the premise of small trauma and retaining thyroid function, while the latter has large trauma, wide resection and high complication risk. It has been reported that the recurrence rate of surgical resection is 29% to 42%, and preoperative volume of contralateral lobe and weight of resected thyroid gland can be important predictors of recurrence 30, 31. And inadequate surgery is also one of the important factors for thyroid nodule recurrence. In addition, hypothyroidism is related to the method of excision, and hypothyroidism is obvious in patients with total thyroidectomy 31. In contrast, RFA was better at preserving thyroid function in patients. Levothyroxine supplementation was not required in 28.3% of RFA patients one year after surgery 32. Therefore, few studies use thyroid function as a predictor of recurrence. Nevertheless, RFA is an acceptable and effective treatment without increasing the risk of complications.

On the one hand, our study mainly has the following limitations: this research is a single-center retrospective analysis, with a small sample and possible sample bias. The conclusion is not representative. A multi-center and larger number of sample studies should be carried out to get more comprehensive evidence-based evidence. On the other hand, the short follow-up time of patients cannot predict the long-term disease recurrence of patients. Long-term follow-up should be carried out in the next study to further clarify the risk factors for disease recurrence after RFA treatment.

For patients with thyroid nodules, it is an important measure to improve the prognosis and reduce the economic burden of patients to improve the ability to predict postoperative recurrence of nodules and implement personalized prevention programs. This study showed that WNS, ND, DRN and recurrent laryngeal nerve injury were independent risk factors for the recurrence of thyroid nodules after radiofrequency ablation, and the combined model had high predictive value. Using this model, patients at high risk of recurrence can be screened as early as possible, so that they can be focused on and preventive measures can be taken.
